# Semantic cues modulate brain activity in different Dunhuang narrative murals appreciation: an fNIRS study

**DOI:** 10.1038/s41598-026-47144-y

**Published:** 2026-04-08

**Authors:** Tinghu Kang, Haiyan Wang, Haiyan Wang, Zhen Man, Zhongping Shi, Bailing Li

**Affiliations:** 1 School of Psychology, Northwest Normal University, 967 Anning East Road, Lanzhou City, Gansu Province China; 2 Acadmy of Fine Art & Design, Northwest Normal University, 967 Anning East Road, Lanzhou City, Gansu Province China; 3 Jining Senior Vocational School, No. 8, Jidai Road, Rencheng District, Jining City, Shandong Province China

**Keywords:** Dunhuang murals, Neuroaesthetics, Narrative structure, Semantic cue, fNIRS, Neuroscience, Psychology, Psychology

## Abstract

Dunhuang murals are an important part of China’s cultural heritage. Due to the influence of painting space and painting techniques, they have given rise to various narrative structures. The impact of different narrative structures on viewers’ aesthetic experience still lacks empirical exploration. This study used fNIRS to investigate the influence of semantic cues and narrative structure on aesthetic judgment of Dunhuang narrative paintings. The results showed that compared to other narrative types, participants rated serial paintings higher in both aesthetic comprehension and liking. Semantic cues significantly improved comprehension ratings for both serial and multi-segment narrative paintings. The results of fNIRS revealed that serial narratives significantly activated the frontopolar areas, while multi-segment narratives notably activated the premotor cortex. Compared to the uncued condition, the presence of semantic cues significantly activated the dorsolateral prefrontal cortex, frontopolar areas, premotor cortex, and frontal eye fields. Results revealed that participants rated serial murals higher in both understanding and liking. Semantic cues selectively improved understanding depending on mural type but did not affect liking, and exploratory fNIRS results showed cuing- and mural-related modulations in prefrontal/premotor channels that should be interpreted cautiously.

**Electronic supplementary material:**

The online version of this article (10.1038/s41598-026-47144-y) contains supplementary material, which is available to authorized users.

## Introduction

Aesthetic appreciation is an active cognitive–affective process shaped by both visual properties and a viewer’s prior knowledge^1–3^. While the effect of low-level visual factors have been extensively explored, narrative comprehension and contextual knowledge are especially relevant for culturally rich artifacts in which meaning is distributed across multiple scenes and symbols. Dunhuang story murals provide an ecologically valid case because they vary systematically in narrative organization (e.g., serial vs. multi-segment layouts) and can be supplemented by semantic information that may support comprehension. Accordingly, the present study examines how narrative structure and semantic cues jointly shape aesthetic judgments (liking and perceived understanding) and explores their cortical correlates using fNIRS.

### Narrative features of Dunhuang murals and aesthetic judgement

As an ancient form of visual narrative art, cave murals serve as important vehicles for recording and transmitting culture, much like tapestries, scroll paintings, and contemporary comics^4^. Narrative painting is a term used in Western art history to refer to the category of paintings whose subject matter is similar to that of genre paintings, but with a stronger narrative or storytelling character^5^. Narrative structure in visual paintings refers to the organization of visual elements (e.g., characters, scenes, actions, and symbols) into a coherent sequence through spatial layout to convey a story or event^6^. Unlike textual narratives, narrative structure in visual narratives is usually conveyed through the spatial arrangement of elements that can suggest chronological order, causality, and thematic connections^7,8^. Therefore, narrative structure in visual art emphasizes the spatial layout of visual elements, through which viewers are guided to interpret narrative elements such as time, cause and effect, and theme^9^. Influenced by cave structure, narrative content, and the evolution of painting techniques, the narrative structure of Dunhuang story paintings evolved from subject-based compositions to serial and multi-segment narratives^10^. The subject-based narratives are characterized by a dominant central character around whom various narrative episodes are randomly arranged in space. This style is analogous to fragmented narration in literary works and hinders the viewer’s understanding of the temporal progression of the story. The serial narratives skillfully use elements such as mountains, trees, and buildings to compartmentalize individual story episodes. These unfold sequentially from left to right, similar to contemporary comics where black frames separate each scene. This structure aids viewers in identifying narrative units and inferring story development^10^. The multi-segment narratives are often used in depicting the life of the Buddha, detailing every stage from birth to enlightenment. For example, the biographical narrative in Cave 290 of the Northern Zhou period employs 87 story episodes to depict the life of Śākyamuni. Although each episode has clear boundaries, the segmented layout results in a more complex viewing path, often in an “S” or “Z” shape^11^.

According to the Parallel Interfacing Narrative-Semantics Model (PINS Model)^12^, the comprehension of narrative paintings follows a consistent cognitive process: first identifying story units, then constructing a situation model, and finally making inferences to predict and update that model. This model emphasizes the importance of narrative structure in narrative understanding. Empirical evidence further showed that sequentially organized images, which aligned with habitual cognitive processing, were processed more fluently than scrambled or disordered image sequences^6,9^. Based on these theoretical and empirical foundations, it can be inferred that serial narratives likely facilitate more fluent cognitive processing compared to multi-segment narratives, with central-figure compositions being the least fluent. Cognitive processing fluency during aesthetic experiences is closely linked to aesthetic appraisal^13^, and the processing fluency theory of aesthetics posits that the more fluently a painting is processed, the more positive the aesthetic response^14^. We note that such process-oriented frameworks explain behavioral outcomes but do not specify particular brain regions,accordingly, our neural predictions are exploratory applications of these theories. Later research further suggested that aesthetic experience arises from a distributed neural network^3^. Therefore, we propose the following hypotheses:

#### H1

*Relative to subject-based and multi-segment narrative murals, viewers’ ratings of aesthetic understanding and liking for serial narrative murals are higher*.

#### H2

*Narrative structure will modulate task-evoked cortical responses in prefrontal and premotor/attentional regions during mural viewing, consistent with differences in narrative integration and control demands. Given the exploratory nature and spatial constraints of fNIRS, we do not advance anatomically fine-grained predictions beyond this process-level expectation*.

### Semantic cues and aesthetic judgement

According to the subjectivist view of artistic aesthetics^2^, aesthetic judgments are influenced not only by the visual characteristics of an artwork but also by the viewer’s knowledge background^15–17^. The model of aesthetic appreciation and aesthetic judgments proposes that aesthetic judgment is one of the outcomes of a series of cognitive and emotional processes, wherein the stage of cognitive mastery is primarily shaped by the individual’s knowledge and experience^18^. Extensive behavioral research has also shown that semantic cues can significantly enhance viewers’ comprehension of paintings^15,19,20^, though their effect on aesthetic judgements such as aesthetic liking remains controversial (e.g., some studies report no effect, while others suggest more positive emotional reactions)^17,21^. Neuroaesthetic studies further reveal that semantic cues influence the activation of brain regions in the process of aesthetic judgment^22–24^. For example, Kirk^24^ found that pleasure ratings were higher for art gallery pieces and that these pieces activated the medial orbitofrontal cortex and prefrontal cortex. When the paintings were primed with the word “fake”, the functional connectivity between the occipital and frontal lobes was enhanced^23^.

Building upon this foundation, we posit that semantic cues may play a particularly crucial role in the aesthetic appreciation of culturally rich but potentially unfamiliar art forms like the Dunhuang murals. By providing essential narrative context, semantic cues are expected to facilitate cognitive mastery, a process theoretically associated with the engagement of prefrontal regions involved in semantic reasoning and knowledge integration^25^. Furthermore, according to the stopping for knowledge hypothesis, successful reduction of prediction error during aesthetic appreciation is associated with a shift in neural activity, potentially involving the deactivation of effort-related regions such as the premotor cortex (BA6) and the enhanced activation of reward-related or knowledge-integration pathways^26^.Therefore, we hypothesize that for Dunhuang murals:

#### H3

*Providing semantic cues will improve viewers’ aesthetic understanding, particularly for murals with clearer narrative organization, and will modulate task-evoked activity within networks supporting semantic integration and cognitive control (e.g., prefrontal nodes). Given the exploratory nature and spatial constraints of fNIRS, we do not advance directional, region-specific predictions for motor/premotor channels*.

## Methods

### Participants

To guarantee sufficient statistical power and avoid underestimation of effects, an a-priori power analysis was conducted with G*Power 3.1^27^. The analysis targeted the interaction effect in a mixed-design ANOVA with two between-subject groups and three repeated-measurement levels. With *α* = 0.05, effect size *f* = 0.25, power = 0.80, correlation among repeated measures = 0.5, and non-sphericity correction = 1, the minimum required total sample size was 28, yielding an actual power of 0.81. To account for potential data loss (e.g., motion artifacts or incomplete responses), we recruited 42 participants; after excluding five participants due to invalid or incomplete data, 37 participants were included in the final analyses. We note that this power analysis was based on behavioral effects, the power for neural effects (with 63 channel-wise comparisons) is inherently lower, so our fNIRS findings should be interpreted as exploratory. Artistic interest was assessed prior to the experiment using a brief self-report questionnaire (15 items, 7-point Likert scale; total score range: 31–74; higher scores indicate greater interest in visual art)^28^. Baseline equivalence between the uncued and cued groups was tested using an independent-samples t-test (reporting Cohen’s d). No significant group difference was observed (*M*__uncued_ = 53.68, *SD* = 11.27; *M*__cued_ = 55.50, *SD* = 9.05; *t*(35) = − 0.538, *p* = 0.426, *d* = − 0.177).

Each participant was compensated with 50 CNY (approximately $7) after completing the experiment. Before the commencement of the research, participants were thoroughly informed about the study’s purpose, procedures, potential risks and benefits, and their right to withdraw at any time. The research team ensured that all participants fully understood experimental procedure. All participants filled out an informed consent form before the experiment began. This study adhered to relevant ethical standards and guidelines. And this study received ethical approval from the Ethics Committee of Scientific Research Projects at the School of Psychology, Northwest Normal University, under approval number 2023109.

### Experimental materials

The photo materials were sourced from the official website of “Digital Dunhuang,” featuring 21 Dunhuang story paintings. These materials are publicly accessible and may be utilized for experimental research purposes. Two experts were invited to categorize these paintings into three types based on narrative structure: subject-based, multi-segement, and serial murals, with seven paintings in each category. To obtain the story content of these experimentally selected paintings, textual materials were gathered from *The Dictionary of Dunhuang Art* by Fan^11^, as well as from Xie’s works on Dunhuang mural paintings of causal stories, *Buddhist stories and Bunsen stories*^29–31^.

Based on Glaser and Schwan^32^, each semantic cue was organized into four parts: (1) the painting’s name, (2) the historical period, (3) the event or story depicted, and (4) a description of the painting’s narrative unit^32^. We derived these details from authoritative sources^11,29–31^ to ensure accuracy. Each cue was approximately 200–220 words long. The readability of all cues was high (LIX scores 46.3–60 out of 100, indicating easy comprehension). For full transparency, the complete texts of all semantic cues used in the experiment are provided in Supplementary Material Appendix 1.

### Experimental procedure

The experiment consisted of two phases: a viewing phase and an evaluation phase. Participants were randomly divided into two groups (uncued vs. cued condition). In the uncued condition, participants directly viewed each mural for 10 s (free browsing), then proceeded to the evaluation phase. In the cued condition, participants first read a textual semantic cue about the mural’s story for 30 s, then viewed the mural for 10 s. In both conditions, the viewing phase was followed by an evaluation phase where participants answered two questions: one rating their liking of the painting and one rating their understanding of it (each on a 7-point scale).

**Fig. 1:**
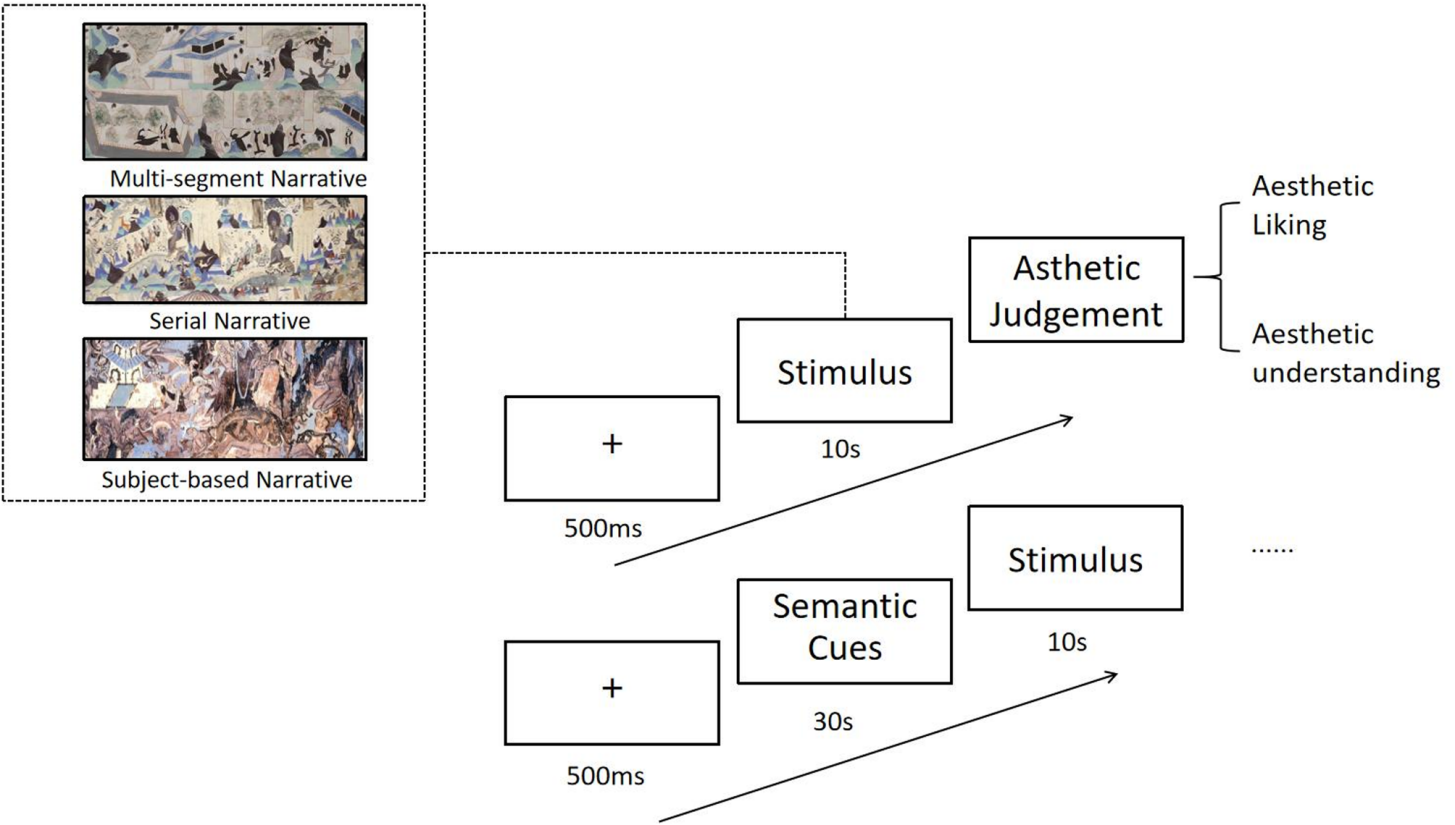
Experimental procedure for uncued and cued conditions.

In the uncued group, subjects browsed the Dunhuang murals directly for 10 s, and then pressed the ‘space bar’ to proceed to the next screen. (This self-paced pause ensured that participants moved on to the questions only after they finished viewing the image.) After pressing the space bar, two aesthetic judgment questions were presented on separate screens, with only one question presented on each screen, the two aesthetic judgment items were: “Please rate your degree of liking/understanding of this painting on a scale from 1 to 7,” where “1” indicates “strongly dislike/do not understand” and “7” indicates “strongly like/understand.” The subjects tried to complete the aesthetic judgment task by hitting the corresponding numbers in the keyboard. After pressing a key, participants saw a white screen for 10 s to collect resting state data. Under the cued condition, they first viewed an introduction to the story content of each Dunhuang mural for 30 s. Subsequently, they observed the mural for 10 s and completed an aesthetic judgment task. The experiment was structured as a long trial, comprising 21 formal trials and 2 practice trials, with a duration ranging from 25 to 35 min (Fig. 1).

*Uncued condition*—participants viewed each mural for 10 s with no prior information, then rated their liking and understanding. Bottom: *Cued condition*—participants first read a semantic cue (30 s) about the mural’s story, then viewed the mural for 10 s, followed by the same liking and understanding ratings. A 10 s rest period occurred after the ratings (not shown) before the next trial.The three Dunhuang murals depicted in the figure are sourced from the Dunhuang Research Academy.

### Data collection and processing

#### Data recording

The experiments were performed using the fNIRS Smart-6000A device (Danyang Huichuang Medical Equipment Co., Ltd., Jiangsu, China). Changes in brain oxyhemoglobin (HbO) and deoxyhemoglobin (HbR) concentrations were continuously measured and recorded as subjects performed the task. The system consists of a near-infrared light source (light-emitting diode, LED) and an avalanche photodiode (APD) as a detector, and the light source probes are used at wavelengths of 730 nm and 850 nm, respectively, and at a sampling rate of 11 Hz. The experiment employs 24 light-source probes and 24 detector probes, which constitute 63 effective channels with an average distance between the emitters and detectors of 3 cm (range 2.7–3.3 cm). Based on the probe geometry and alignment with standard brain anatomy, we identified the likely cortical regions for each fNIRS channel (see Table 1). Table 1 lists each channel alongside its approximate Brodmann area and the percentage overlap of its sensitivity profile with that region. Based on previous studies, the main brain regions observed in this study were the frontal and parietal lobes^33^.

**Table 1:** Spatial alignment information for the relevant fNIRS channels in the experiment.

channel	Transmitter-detector	MNI coordinates			Brodmann partitioning and brain region overlap*
		x	y	z	
CH5	S3-D2	40	63	− 3	10—Frontopolar area(0.91)
CH7	S3-D8	30	67	13	10—Frontopolar area(1)
CH29	S11-D23	− 51	20	44	9—Dorsolateral prefrontal cortex(0.49)
CH36	S14-D18	− 37	− 3	65	6—Pre-Motor and Supplementary Motor Cortex(1)
CH39	S14-D23	− 43	19	55	8—Includes Frontal eye fields(0.78)
CH46	S17-D17	56	− 4	52	6—Pre-Motor and Supplementary Motor Cortex(0.77)

*Some fNIRS channels cover multiple brain regions. To conserve space, only the region with the highest overlap is listed for each channel in this table.

#### Data analysis

The fNIRS Spark software (HuiChuang, China) was used for data preprocessing. First, the raw optical density data were corrected for motion artifacts using a wavelet-based motion de-artifacting method^34^, Second, the data were band-pass filtered using a 0.01–0.2 Hz filter; Finally, the filtered optical density data were converted to the concentration change data Δ[HbO] and Δ[HbR] of the oxidized and deoxidized hemoglobin HbO and deoxygenated hemoglobin HbR based on the modified Beers-Lambert law. In this study, because Δ[HbO] has a higher signal-to-noise ratio than Δ[HbR] and is more sensitive to changes in cerebral blood flow^35^, subsequent statistical analyses were performed using Δ[HbO] data.

A general linear model (GLM) was used to solve the task-related *β*-values under different conditions, and the *β*-values were used as indicators of activation in the corresponding brain regions^36^. Notably, the button-press occurred at the end of the viewing phase, and a 10 s rest period followed each trial, our GLM timing (with the 10 s trial window and pre-stimulus baseline) was set such that the primary analysis window for stimulus-related activation did not overlap with the subsequent motor response. A long trial design was used, with 200 ms before the appearance of the picture stimulus as the baseline period and 10 s after the picture stimulus as the length of the trial, which was used as a time cue for convolution with HRF. We acknowledge that this baseline is short given the slow hemodynamics of fNIRS; it was chosen to capture the immediate pre-stimulus level, while a 10 s rest after each trial helped the signal return towards baseline.

Statistical analysis was performed using SPSS 26.0 software (IBM, Somers, USA). The significance level was set at 0.05. Descriptive statistics are reported as mean and standard deviation (*M* (*SD*)). The* β*-values for each channel were analyzed by 2 (cue type: uncued, cued) × 3 (mural type: multi-segment, serial, subject-based) repeated-measures ANOVA, with Greenhouse–Geisser correction for sphericity and Bonferroni correction for post hoc multiple comparisons. Finally, the *p*-values were corrected for multiple comparisons between channels using the FDR method to further reduce the false positive rate.

No specific regions of interest were defined a priori; instead, an exploratory channel-wise analysis was performed across all 63 channels. To account for multiple comparisons, we applied a Benjamini–Hochberg false discovery rate (FDR) correction (*q* < 0.05) to the set of channel-wise tests for each effect (cue main effect, mural type main effect, and their interaction). Only channels surviving this correction were considered significant.

## Results

### Aesthetic judgments results

The descriptive statistics of participants’ aesthetic judgment scores when viewing different types of murals under different cuing conditions are shown in Table 2.

**Table 2:** Descriptive statistics of aesthetic judgments when browsing different types of murals under different cuing conditions *M(SD).*

Cuing condition	Types of murals	Understanding rating	Liking rating
Uncued	multi-segment	3.20(1.26)	3.96(1.56)
	serial	4.31(0.94)	4.54(1.46)
	subject-based	4.01(1.13)	4.02(1.32)
cued	multi-segment	4.10(1.25)	4.65(1.14)
	serial	5.00(1.06)	5.18(1.05)
	subject-based	3.74(1.29)	4.38(1.25)

**Fig. 2:**
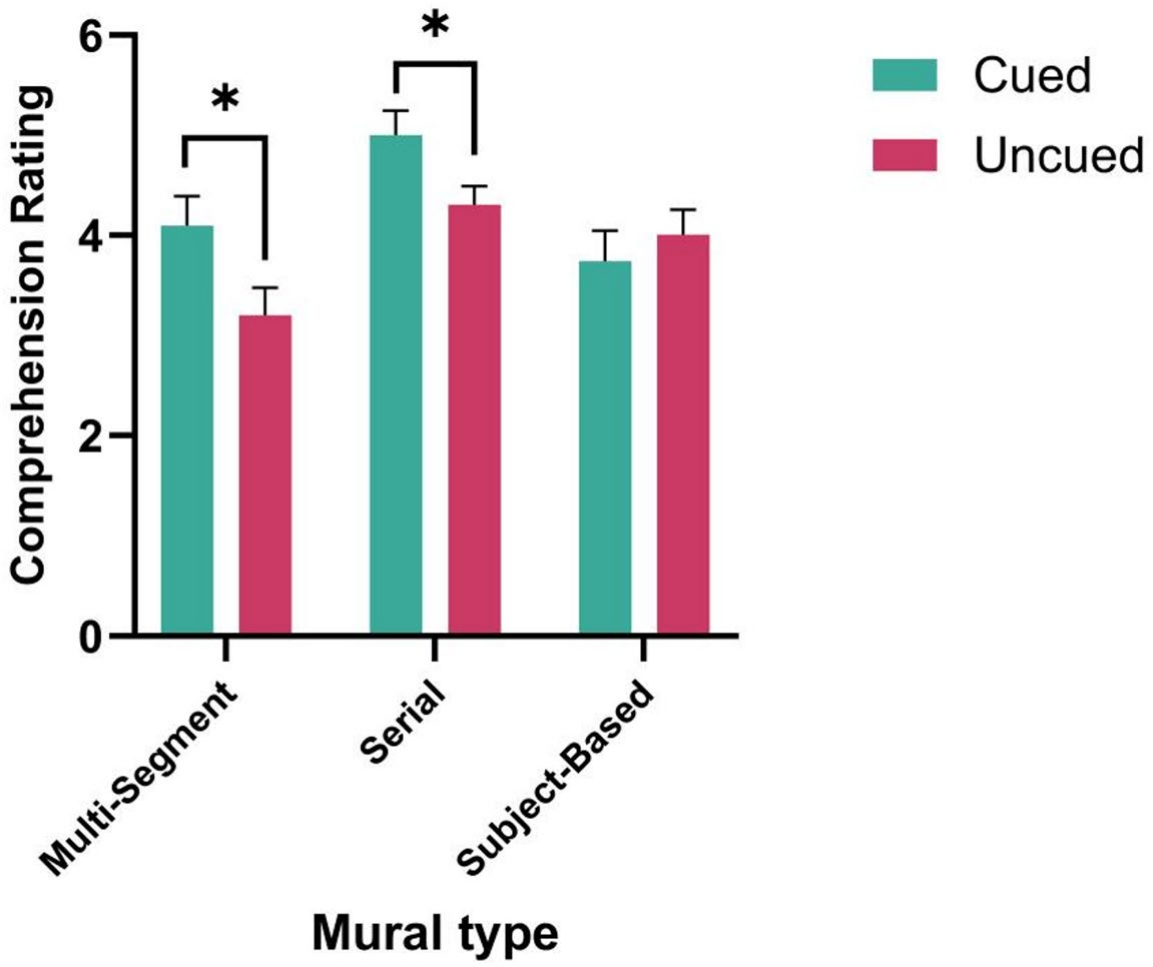
The effect of the semantic cue on comprehension rating for various types of murals.

A 2 (cuing condition: uncued, cued) × 3 (mural type: multi-segment, serial, subject-based) ANOVA was conducted on scores of aesthetic understanding and liking. The results revealed a significant main effect of mural type on the aesthetic understanding rating, *F*(2,70) = 20.692,* p* < 0.001, η_p_^2^ = 0.359. Post hoc multiple comparisons revealed that the understanding rating of serialized murals was significantly higher than multi-segmented paintings (*p* < 0.001) and subject-based murals (*p* < 0.001). The main effect of semantic cues on comprehension was not significant, *F*(1,35) = 1.935, p = 0.173, ηp^2^ = 0.052. The interaction effect between cuing condition and mural type was significant, *F*(2,70) = 7.152*, p* = 0.001, η_p_^2^ = 0.162).This indicates that semantic cues improved comprehension only for certain types of murals (see simple effects), and not uniformly for all mural types. Further simple-effects analysis revealed that when individuals viewed serialized and multi-segmented murals, individuals’ understanding ratings were significantly higher in the cued condition than in the uncued condition (*F*(1,35) = 5.125, *p* = 0.030, η_p_^2^ = 0.122; *F*(1,35) = 4.911, *p* = 0.033, η_p_^2^ = 0.117) (see Fig. 2).

On the aesthetic liking rating, the main effect of mural type was significant, *F*(2, 70) = 8.285, *p* = 0.001, η_p_^2^ = 0.183. Post-hoc multiple comparison analyses revealed that individuals’ liking ratings of serialized murals were significantly higher than multi-segmented (*p* = 0.022) and subjective murals (*p* = 0.002). None of the other effects were significant.

### fNIRS results

A 2 (cuing condition: uncued, cued) × 3 (mural type: multi-segment, serial, subject-based) repeated-measures ANOVA was conducted on 63 whole-brain channels.

**Fig. 3:**
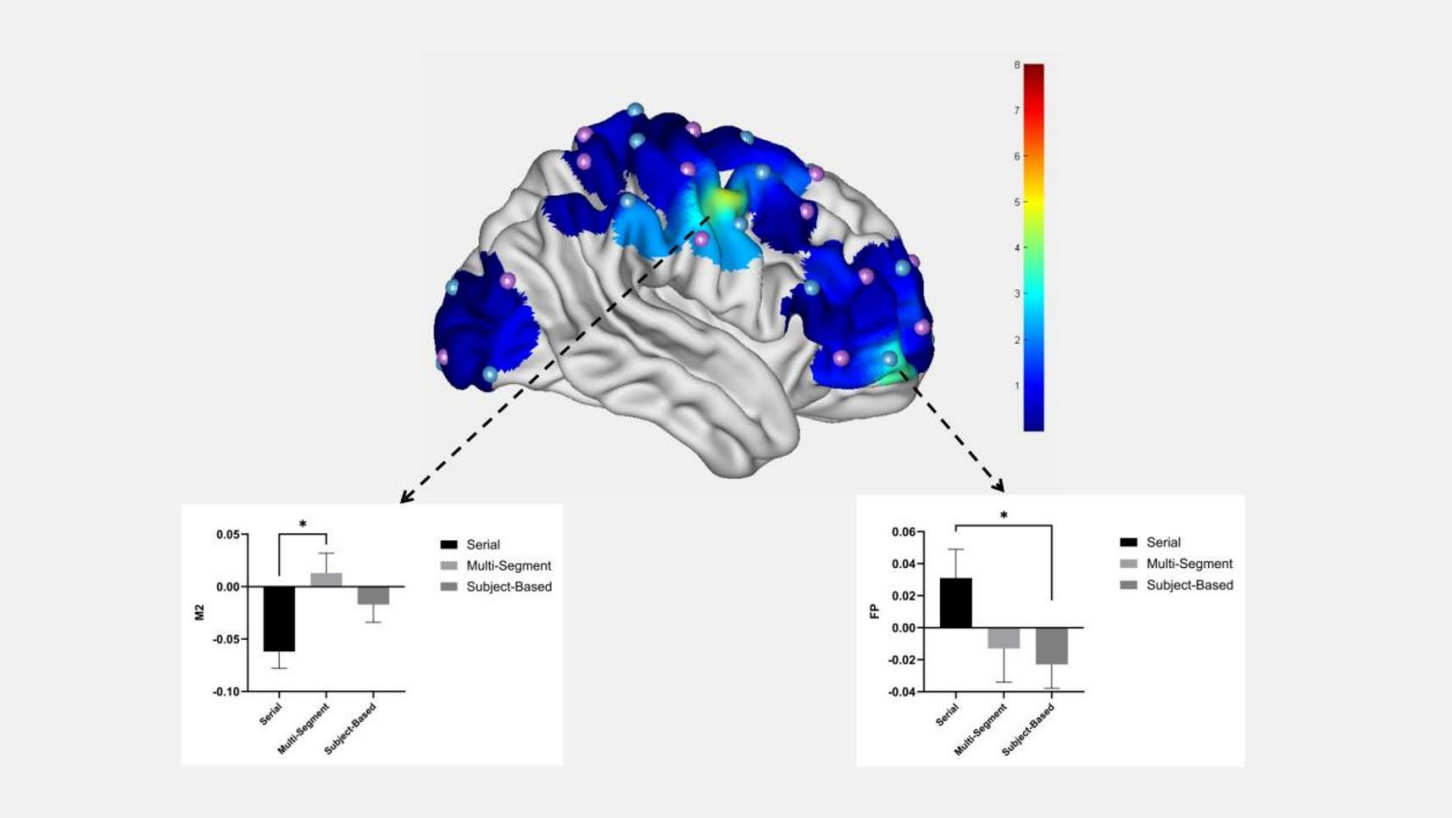
Effects of the mural type in the M2 and FP area.

The main effect of mural type was significant in channels 5 and 46, the descriptive statistics are presented in Table 3. Post hoc multiple comparisons revealed that the right frontal pole (channel 5) had the highest level of activation when processing serial murals, which was significantly higher than subject-based murals (*p* = 0.020). The right premotor cortex (BA6) areas (channel 46) had the highest activation flattest when processing multisegmental murals, significantly higher than serial murals (*p* = 0.045) (see Fig. 3).

**Table 3:** Results of the mural type main effects.

Channel	Brain area	*F*-value	*p*-value	Multi-segment β-value	Serial β-value	Subject β-values
5	Frontal pole	3.616	0.032	− 0.013 ± .021	0.031 ± .018	− 0.023 ± .015
46	Premotor cortex	4.801	0.011	0.013 ± .019	− 0.062 ± .016	− 0.017 ± .017

**Fig. 4:**
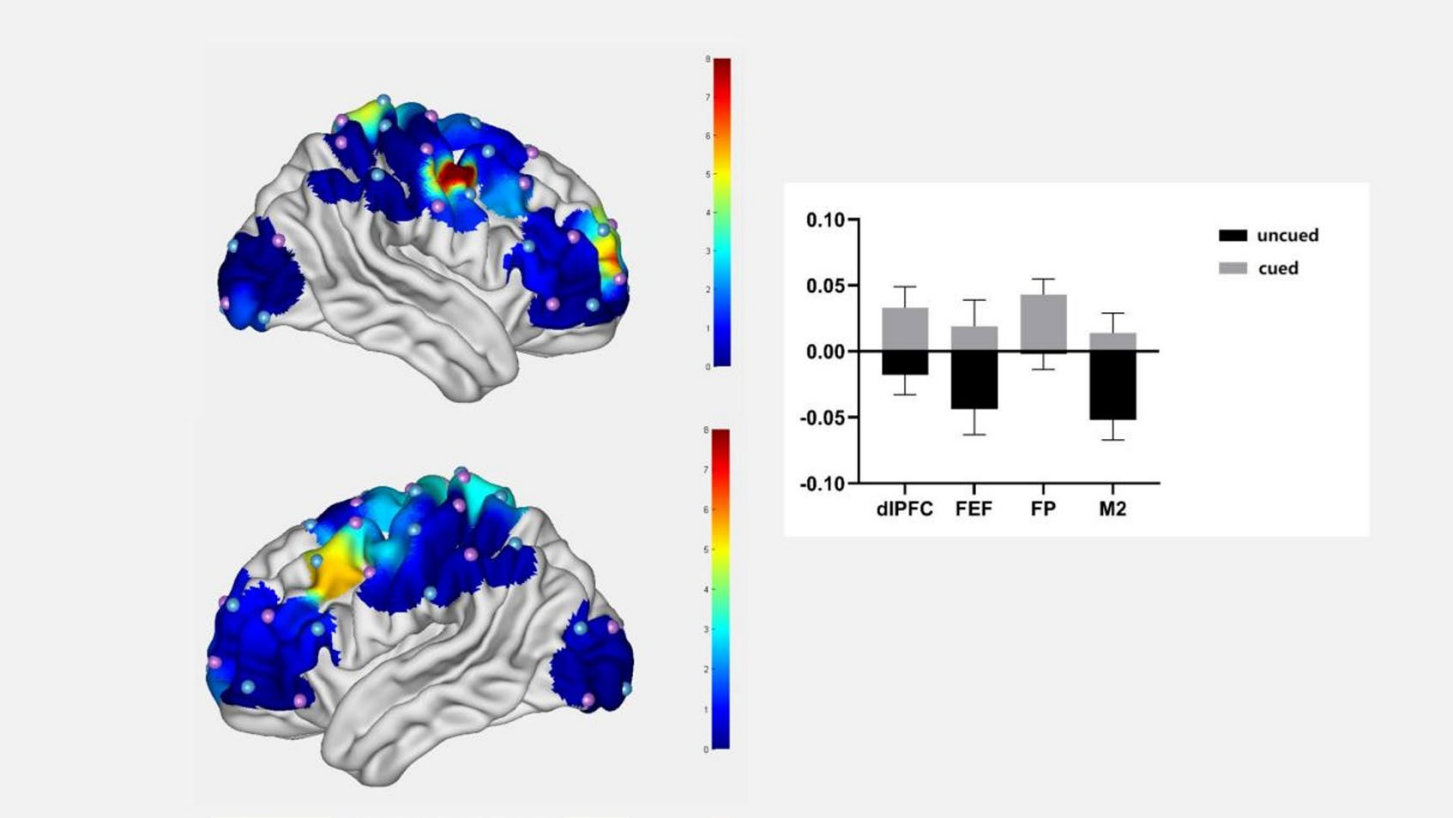
The effects of the cuing condition on the relevant brain areas.

The main effect of the cuing condition was significant in channels 7, 29, 39, and 46, the descriptive statistics are presented in Table 4. The activation levels of these areas were significantly higher in the cued condition than in the uncued condition (right FP: *p* = 0.014; DLPFC: *p* = 0.025; left FEF: *p* = 0.029; right M2: *p* = 0.002) (see Fig. 4).

**Table 4:** Results of the cuing condition main effects.

Channel	Brain area	*F*-value	*p*-value	Uncued β-value	Cued β-value
7	Right FP	6.738	0.014	− 0.002 ± 0.012	0.043 ± 0.012
29	Left DLPFC	5.467	0.025	− 0.018 ± 0.015	0.033 ± 0.016
39	Left FEF	5.198	0.029	− 0.044 ± 0.019	0.019 ± 0.020
46	Right M2	11.332	0.002	− 0.058 ± 0.015	0.014 ± 0.015

Frontal Pole, FP; Dorsolateral Prefrontal Cortex, DLPFC; Frontal Eye Field, FEF; Pre-Motor and Supplementary Motor Cortex, M2.

**Fig. 5:**
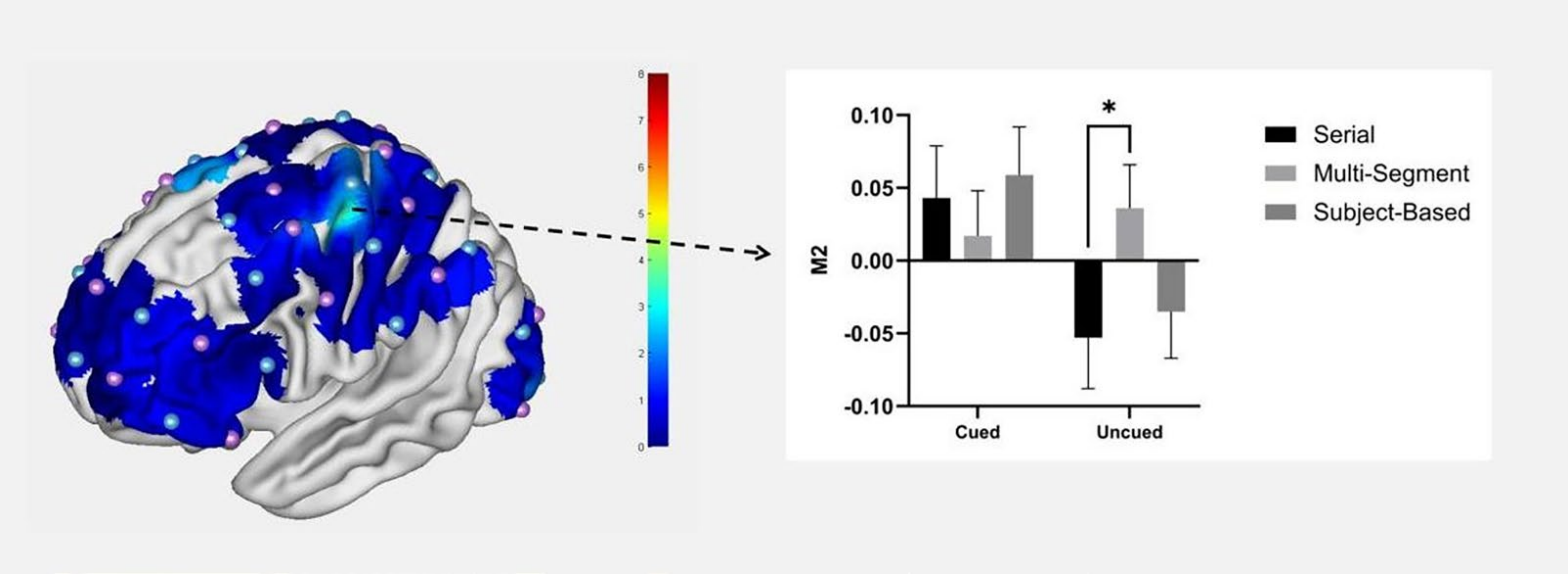
Interaction between mural type and cuing condition in the M2 area.

There were significant interaction effects between cuing condition and mural type in left M2 (channel 36)(F(2, 70) = 3.876, *p* = 0.025, η_p_^2^ = 0.100). Further simple effect analysis revealed that in the uncued condition, the activation level of M2 brain area when participants viewed the multi-segment murals was significantly higher than viewed serial murals(*p* = 0.036) (see Fig. 5).

## Discussion

The present study investigated the neural mechanisms through which semantic cues and narrative structure influence aesthetic judgments of Dunhuang murals. Behaviorally, participants both preferred and understood serial murals better than multi-segmented and subject-based ones, providing support for H1. Furthermore, semantic cues significantly enhanced viewers’ comprehension of multi-segmented and serial murals (but not the subject-based murals), which partially supports H3. This suggests that semantic cues aid understanding primarily for narratively complex paintings, rather than exerting a universal effect across all mural types. The fNIRS imaging revealed that both mural types and semantic cues engaged the right frontal pole (BA 10) and the premotor cortex (BA6). Additionally, semantic cues alone activated the dorsolateral prefrontal cortex (BA 9) and the frontal eye fields (BA 8). An interaction effect between mural type and semantic cues was observed in the the premotor cortex (BA 6), consistent with H2 and H3. These findings are in line with theoretical models (e.g., stopping-for-knowledge hypothesis) that propose cognitive engagement during art viewing involves both knowledge acquisition and motor-preparatory processes. However, given BA6’s diverse functions (spanning premotor and supplementary motor areas), we interpret the BA6 activation in our study as reflecting a general engagement of motor-related networks (perhaps attention-orienting or imagery) rather than a specific “exploratory” drive. In other words, viewers may not need to “mentally simulate” as much movement when the narrative is clear (serial murals), leading to lower BA6 activation.

### Neural responses to mural narrative structures

The main effects of mural type were observed in the right frontal pole and premotor cortex. Specifically, relative to multi-segmented and subject-based murals, serial murals elicited reduced activation in the right premotor cortex compared with baseline, while the right frontal pole (BA10) was markedly activated. Both regions are known to play a role in aesthetic judgment, as part of distributed networks rather than in isolation. These findings are consistent with the stopping-for-knowledge hypothesis and network-based models of aesthetic experience^3^, suggesting that narrative structure and semantic cues engage partially distinct but interconnected neural networks during the aesthetic evaluation of Dunhuang murals. For example, the frontal pole activation can be viewed in the context of a default-mode or knowledge-evaluation network, whereas the premotor cortex (BA6) activation likely reflects involvement of attentional-motor networks, together they indicate a network-level interaction between cognitive evaluation and attentional orienting. We caution that this decrease is relative to a short baseline and its exact physiological meaning (e.g., neural deactivation vs. hemodynamic undershoot) is uncertain.

Furthermore, the frontal pole demonstrated significantly higher activation during the viewing of serial murals compared to other narrative structures. Previous research has linked the frontal pole to knowledge discovery during visual art appreciation^37–39^. A meta-analysis on the neural correlates of aesthetic judgment further propose that frontal pole activation reflects the acquisition of new knowledge^26^. This idea resonates with the long-standing view, dating back to Aristotle^40^, that aesthetic experience constitutes a form of learning and reasoning.

The brain derives intrinsic reward when it perceives a reduction in predictive uncertainty over time. Such reward motivates individuals to seek out perceptual experiences that resolve ambiguity while avoiding either overly predictable or chaotic stimuli^41,42^. Aesthetic pleasure arises when viewers successfully integrate sensory input and minimize prediction error. Consequently, the more comprehensible narrative in serial murals enhances aesthetic satisfaction by facilitating knowledge acquisition.

### Effects of semantic cues on neural processing and attention

We observed that the presence of semantic cues during mural appreciation elicited significant activation across multiple brain regions, including the frontal pole, premotor cortex (BA6), dorsolateral prefrontal cortex (DLPFC) and frontal eye fields. This pattern suggests that semantic cues induce synergistic neural responses involving both cognitive and attentional systems.

These findings are consistent with previous research indicating that semantic enrichment in artworks modulates prefrontal activity, which plays a central role in aesthetic judgment^22,23^. Specifically, compared to the uncued condition, semantic cues led to significantly stronger activation in the premotor cortex (BA6) and frontal pole. This enhanced neural response suggests that semantic cues deepen cognitive engagement and facilitate knowledge acquisition^39^, possibly by increasing attentional allocation and promoting conceptual integration. Notably, activation of the DLPFC which a region implicated in inferential reasoning and meaning negotiation during aesthetic experience, and provides further evidence that semantic cues support structured knowledge building and enhance viewers’ reasoning processes while interpreting the murals.

Additionally, cued conditions were associated with significantly enhanced activation in the frontal eye fields, a region central to attentional orienting and presaccadic preparation. This area contributes to top-down attentional control by enhancing visual sensitivity at relevant locations and suppressing irrelevant ones during eye movement planning^43^. Such activation suggests that semantic cues guide viewers’ visual exploration, reduce ocular dynamic entropy, and streamline attentional focus toward meaningful narrative elements.

In summary, while mural narrative type primarily engaged regions related to general cognitive processing, semantic cues recruited a broader network associated with both emotional and high-level cognitive functions. These results indicate that semantic cues enhance attentional engagement, facilitate knowledge acquisition, and promote emotional resonance during the aesthetic experience of Dunhuang murals.

Our neural findings, though statistically significant in some channels, were modest in magnitude (small β-value differences) and spatially limited to isolated channels. We interpret these results cautiously, as tentative neural correlates of narrative processing, rather than as evidence of any broad or robust neural activation pattern.

### Theoretical contributions

This study contributes to neuroaesthetics in three ways. First, it extends neuroaesthetic research to an ecologically valid and culturally rich stimulus set—Dunhuang story murals—while systematically manipulating narrative organization. Second, it demonstrates a dissociation between perceived understanding and aesthetic liking, consistent with multi-stage accounts of aesthetic experience, and shows that semantic cues support comprehension in a manner that depends on narrative structure. Third, it provides exploratory fNIRS evidence suggesting that cue-supported appraisal and narrative organization are associated with modest differences in prefrontal and premotor/attentional cortical responses. These preliminary neural correlates motivate future work with designs and modalities better suited to testing network-level mechanisms.

### Limitations

However, this study has several limitations. Statistically, although the sample size was powered for behavioral effects, the exploratory fNIRS analyses (many channels/comparisons) likely had limited power to detect small neural differences, so some effects may have been missed. Methodologically, the spatial resolution of fNIRS constrains precise interpretation, especially for small or deep regions. This long-trial sequence constrains stage-specific inference; viewing-related fNIRS signals likely capture a mixture of comprehension and evaluative processing rather than a single isolated component.

In addition, we conducted an exploratory, whole-channel fNIRS analysis without predefined ROIs; despite FDR correction, some findings may still be false positives and should be interpreted cautiously until replicated. Moreover, the GLM used a very brief 200-ms pre-stimulus baseline, which may not capture slow hemodynamic trends and complicates interpretation of negative-going responses. Finally, sample representativeness and differences between laboratory and real-world art appreciation may limit generalizability and ecological validity; the results are correlational rather than causal, and potential moderators (e.g., expertise, cultural background) and the specific nature of emotional experience were not examined in depth. Future work should adopt more ecological designs with diverse samples and interventional approaches to further validate and extend these findings.

## Conclusion

This study offers initial insights into how narrative structure and semantic cues may engage the brain during the aesthetic appreciation of Dunhuang murals. Our neural results point to distinct patterns of activation (frontal vs. premotor) associated with different narrative experiences, suggesting potential neural correlates of cognitive understanding and attentional engagement. However, these patterns are exploratory and require further confirmation.

Overall, our findings contribute to a growing understanding of the neural basis of aesthetic processing in visual arts, while underscoring the need for more research to draw definitive conclusions. They also provide a scientific perspective that can inform the conservation and presentation of Dunhuang murals, emphasizing the importance of narrative context in viewer experience.

## Electronic supplementary material

Below is the link to the electronic supplementary material.

Supplementary file 1

## Data Availability

The original contributions presented in the study are included in the article, further inquiries can be directed to the corresponding author.

## Code availability

All preprocessing and statistical analyses were conducted using fNIRS Spark and SPSS 26.0. No custom code or mathematical algorithms were developed in this study. Analysis settings and output logs are available from the corresponding author upon reasonable request.
