# Supplementary material for: Semantic cues modulate brain activity in different Dunhuang narrative murals appreciation: an fNIRS study
Source: Sci Rep. 2026 Apr 8;16:16644. doi: 10.1038/s41598-026-47144-y (PMC13219484; doi:10.1038/s41598-026-47144-y)
Supplement: Supplementary file 1 [file 41598_2026_47144_MOESM1_ESM.docx]

**Appendix 1: Example of Semantic Cues**

Mogao Cave 254, south wall, Northern Wei dynasty,“Jātaka of Prince Sārdūla.”

The mural recounts how Prince Sārdūla gave his life to save starving tigers. The episodes unfold as follows: three princes go hunting and find an emaciated tigress with her cubs. Sārdūla resolves to sacrifice himself; he climbs a cliff, leaps down, and offers his body to the tigers. After feeding on his flesh the tigers regain their strength. His two elder brothers discover the bones, grieve deeply, and rush to inform their parents. The king and queen, overwhelmed with sorrow, embrace their son’s remains and later erect a stūpa to commemorate his selfless virtue.

(Chinese character count: 219)
